# Disparities in Premature Mortality Between High- and Low-Income US Counties

**Published:** 2012-03-22

**Authors:** Erika R. Cheng, David A. Kindig

**Affiliations:** Department of Population Health Sciences, School of Medicine and Public Health, University of Wisconsin – Madison; Department of Population Health Sciences, School of Medicine and Public Health, University of Wisconsin – Madison, Madison, Wisconsin

## Abstract

**Introduction:**

Several well-established determinants of health are associated with premature mortality. Using data from the 2010 County Health Rankings, we describe the association of selected determinants of health with premature mortality among counties with broadly differing levels of income.

**Methods:**

County-level data on 3,139 US counties from the 2010 County Health Rankings were linked to county mortality data from the Centers for Disease Control and Prevention Compressed Mortality database. We divided counties into 3 groups, defined by sample median household income levels: low-income (≤25th percentile, $29,631), mid-income (25th-75th percentile, $29,631-$39,401), and high-income (≥75th percentile, ≥$39,401). We analyzed group differences in geographic, sociodemographic, racial/ethnic, health care, social, and behavioral factors. Stratified multivariable linear regression explored the associations of these health determinants with premature mortality for high- and low-income groups.

**Results:**

The association between income and premature mortality was stronger among low-income counties than high-income counties. We found differences in the pattern of risk factors between high- and low-income groups. Significant geographic, sociodemographic, racial/ethnic, health care, social, and behavioral disparities exist among income groups.

**Conclusion:**

Geographic location and the percentages of adult smokers and adults with a college education were associated with premature mortality rates in US counties. These relationships varied in magnitude and significance across income groups. Our findings suggest that population health policies aimed at reducing mortality disparities require an understanding of the socioeconomic context within which modifiable variables exist.

## Introduction

Increasing attention is being drawn to the variation in health outcomes across counties in the United States, most recently with the national County Health Rankings (www.countyhealthrankings.org). The population health model underpinning the rankings reflects a multiple determinants of health perspective, with emphasis on health behaviors, socioeconomic status, health care, and the physical environment. This perspective implies that health improvement and disparity reduction will require a balanced investment strategy across multiple determinants of health.

Income is well-recognized to be associated with morbidity and premature mortality internationally and within the United States (1,2). Several mechanisms may explain this relationship. Poverty is thought to affect health through material deprivation, decreased social participation, and decreased control over one's life (2). In contrast, health outcomes of people with higher incomes may be related to increased social participation and control and access to safe neighborhoods, healthy foods, education, health care, and clean air (2).

The relationship between income inequality and health, on the other hand, remains controversial (3,4). Income inequality is associated with low levels of trust and social capital, mental illness, teenage birth rates, low social mobility, homicide rates, obesity, poor educational performance, and mortality (5), and may alter biologic functioning via stress processes (6,7). Other studies, however, report no association between income inequality and mortality disparities after controlling for education (8) or race (9), although these results have been disputed (7).

Increasing evidence suggests that the relationship between income and mortality is nonlinear, with the protective effects of individual income on health diminishing at higher income levels (10-13). In addition, risk factors other than income most likely affect the income-mortality relationship at the county level.

Given this background, the purpose of this study was to examine the relationship between income and premature mortality in US counties and to determine the relative association of several well-established determinants of health and premature mortality among counties with broadly differing levels of income. Our examination used county-level aggregate measures of income and mortality. We hypothesized that 1) there would be a nonlinear relationship between income and premature mortality among US counties and 2) factors associated with premature mortality on a national level would have differential effects in low-income and high-income counties.

Understanding these relationships illuminates areas where resources can be targeted toward strategies that will extend years of life. In addition, policies aimed at modifying factors associated with poor health outcomes may need to include consideration of the larger socioeconomic context within which these variables exist.

## Methods

### Study design

We used weighted multivariable linear regression to analyze the association of several well-established determinants of health with premature mortality in US counties. The analyses used data from 3 sources: the 2010 County Health Rankings, the Centers for Disease Control and Prevention (CDC) Compressed Mortality database, and the 2000 Decennial Census.

### Data and sample information

Our sample consisted of all 3,139 counties in the United States. Most of the data on the multiple determinants of health are from the 2010 County Health Rankings (www.countyhealthrankings.org). The County Health Rankings compiles data from several national sources to rank each county within the 50 states by health outcomes and the multiple factors that determine health.

Mortality data for the sample were from the CDC Compressed Mortality database (http://wonder.cdc.gov/wonder/). Mortality data were downloaded by place of residence (US county) and averaged over 5 years (2002-2006) to improve the stability of the estimates. Sociodemographic and descriptive characteristics were from the 2000 Decennial Census (www.census.gov/main/www/cen2000.html).

### Measures

**Dependent variable: premature mortality**

Premature mortality was defined as the all-cause, age-adjusted mortality rate per 100,000 population for all individuals aged birth to 75 years, averaged from 2002 through 2006. Our measure of premature mortality was thus based on all deaths occurring before the age of 75, which is consistent with methodology employed in the County Health Rankings. We measured premature mortality, rather than overall mortality, to underlie the intent of the County Health Rankings to focus attention on deaths that can be prevented.

**Independent variables**

We selected covariates on the basis of previous literature (14) and included the following county-level variables: income (median household income, income inequality), geography (geographic region, population density), race/ethnicity (percentage white, percentage black, percentage nonwhite/nonblack race, percentage Hispanic), health care access and quality (percentage of adults aged less than 65 without insurance, number of primary care providers per 100,000 population, preventable hospitalizations per 1,000 population), socioenvironmental factors (average high school freshman graduation rate per 1,000 population, percentage of adults aged 25 or older with a 4-year college degree, percentage of single-parent households, percentage of children living below federal poverty guidelines), and behavior factors (percentages of adult obesity and smoking).

Income inequality was measured by the Gini index, a score between 0 to 100, with 0 representing complete equality and 100 representing complete inequality in resources. Geographic region was coded as Northeast, Midwest, South, or West (www.census.gov/geo/www/us_regdiv.pdf). Population density, measured in people per square mile, was coded into quartiles: <17, 17-43, 43-104, and >104. High school graduation rate was defined as the percentage of the 9th-grade cohort that graduate from high school in 4 years.

### Statistical methods

We stratified counties into 3 groups representing low-, high-, and mid-income groups defined using cut points based on the 25th and 75th percentiles of county median annual household income in our sample: 1) low-income counties (LIC)We stratified counties into 3 groups representing low-, high-, and mid-income groups defined using cut points based on the 25th and 75th percentiles of county median annual household income in our sample: 1) low-income counties (LIC), <$29,631; 2) mid-income counties (MIC)We stratified counties into 3 groups representing low-, high-, and mid-income groups defined using cut points based on the 25th and 75th percentiles of county median annual household income in our sample: 1) low-income counties (LIC), <$29,631; 2) mid-income counties (MIC), $29,631-$39,401; and 3) high-income counties (HIC), >$39,401.

Analyses were conducted using SAS 9.2 (SAS Institute, Inc, Cary, North Carolina). For the descriptive analysis, we used standard hypothesis tests to assess differences in county-level covariates among income groups. Frequency distributions for noncontinuous variables were compared by using χ^2^ tests of homogeneity. Weighted multivariable regression models were fit to identify factors associated with premature mortality for the sample. Stratified weighted regression analyses focused on the differences in premature mortality determinants between HIC and LIC. Multivariable models were weighted by the log variance of the mortality rates and included an indicator variable for each state.

Some counties were missing values for our explanatory variables. Among the 3,139 counties, 71 were missing information for preventable hospital stays, 70 for average high school freshman graduation rate, and 689 for percentage of adults smoking. To adjust for missing data, we used SAS PROC MI, including all analytic and design variables, to generate 10 complete data sets for all counties and for each income group. We estimated weighted multivariable parameter estimates and robust standard errors within each imputed data set. The final coefficients resulted from SAS PROC MIANALYZE procedures that combined these estimates across data sets.

In addition to the analyses based on multiple imputed data sets, we tested for spatial auto-correlation among counties by means of Moran's *I* statistic based on the first imputed data set by using SAS PROC VARIOGRAM and county latitude and longitude estimates from the US Census Bureau (www.census.gov/geo/www/gazetteer/places2k.html). To aid with interpretation of coefficients, we converted continuous covariates to *z* scores and transformed our dependent variable to a logarithmic scale. β coefficients for continuous covariates thus reflect the percent change in the premature mortality rate associated with each standard deviation (SD) change in the predictor.

## Results

The association between premature mortality and median household income for all 3,139 counties showed the expected negative relationship between income and premature mortality but with variation (Figure). Median household income levels ranged from $9,333 to $82,929 (average, $35,371; SD, $8,916). The average premature mortality rate for the counties was 417 deaths per 100,000 (25th percentile, 345; 75th percentile, 480). A total of 785 counties were classified as LIC, 1,570 as MIC, and 784 as HIC.

**Figure.**
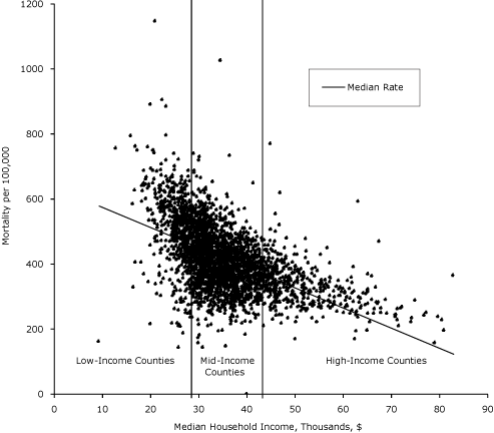
Median annual household income and age-adjusted mortality per 100,000 population aged birth to 75 years, 2002-2006. Bars represent 25th ($29,631) and 75th ($39,401) percentile delineations of median household income for 3,139 US counties. Counties are grouped by median household income levels into low-income (n = 785); mid-income (n = 1,570), and high-income (n = 785) counties.

### Descriptive analysis

Means and SDs for all variables of interest across income groups showed an inverse gradient in median household income levels and premature mortality (Table 1). Decreasing income levels were associated with higher premature mortality rates. Bivariate analyses indicated significant differences in premature mortality between LIC and MIC, and between MIC and HIC (*P* < .001). In addition, significant geographic, sociodemographic, racial/ethnic, health care, social, and behavioral disparities exist among income groups (Table 1).

### Multivariate analysis

In our first model, a 1 SD increase in median household income ($8,916) was associated with a 13% reduction in county-level premature mortality (*P* < .001). Compared with MIC, being classified as an LIC was associated with 18% higher premature mortality and being classified as an HIC was associated with 20% lower premature mortality (*P* < .001 for LIC and HIC).

In a second multivariable regression, the relationship between median household income and premature mortality was attenuated after adjusting for a large number of demographic and sociodemographic characteristics. Among the significant variables, we found reductions in premature mortality rates of 12.5% and 16.4% for the Northeast and Midwest. Geographic location and the percentages of adult smokers and adults with a college education were associated with premature mortality rates in US counties. Using the SD estimates from Table 1, we found that an 8.4% increase in the prevalence of adults with a 4-year college degree was associated with approximately 6% lower premature mortality rates, whereas a 5.9% increase in the percentage of adult smokers increased premature mortality rates by nearly 7% (Table 2).

In a single unadjusted model, the relationship between income and premature mortality was stronger among LIC than among HIC: an increase of 1 SD in county-level median household income was associated with an 18% decrease in premature mortality rates among LICs (*P* < .001) and a 12% decrease in premature mortality rates in HIC (*P* < .001; unadjusted, results not shown).

The results from our stratified multivariable analyses revealed variation in magnitude and significance of our covariates across income groups (Table 3). Although effect sizes varied, increasing percentages of Hispanics and of adults with a 4-year college degree were associated with lower premature mortality rates across all groups. Preventable hospital stays, and the percentages of the population that is black, children living below federal poverty guidelines, and adults smoking were associated with higher premature mortality rates.

Associations varied between strata. Among LIC, the strongest predictor of premature mortality was the percentage of the adult population smoking. For LIC, compared with counties in the West, the Northeast was associated with approximately 19% lower mortality rates. For HIC, the Midwest was associated with more than 17% lower mortality rates than the West. Income inequality was associated with higher premature mortality rates in HIC but was not associated with premature mortality rates among LIC. Among HIC, the percentage black had a stronger positive association with premature mortality rates than among LIC (Table 3). Our test for spatial autocorrelation was not significant (Moran's *I*: *Z* = −0.25, *P* < .001), indicating no spatial autocorrelation in our models.

## Discussion

Consistent with previous research (12-15), we found a nonlinear association between income and mortality among US counties. Specifically, our results show that the association between county-level income and premature mortality is stronger in LIC than in HIC. This association attenuated and lost significance among LIC after adjusting for a wide range of demographic and sociodemographic characteristics.

Our multivariable results illuminate differences in the pattern of determinants between LIC and HIC. For example, the effects of children living below federal poverty guidelines and of single-parent households on premature mortality are stronger among LIC than among HIC, which suggests that county-level income may buffer against the effects of some variables associated with poor health. This buffer may operate through coping resources; county-level income may reflect overall availability of material and social resources that enable affluent single parents access to child care, neighborhood support, social networks, or higher-quality health care (15).

Conversely, our results show that the association between premature mortality rates and some of our covariates was higher in HIC than in LIC, including the percentage of the population that is black. This finding may be related to a more micro-level context: neighborhoods. Regardless of income level, blacks are more likely than whites to live in racially segregated neighborhoods (16). Segregation processes, including exposure to racial discrimination or racial conflict, can affect health through acute and chronic stress (17). Discrimination can restrict socioeconomic opportunities and mobility and affect health through quality of life in neighborhoods, access to and quality of services, and the physical environment (18,19). Perceived discrimination has been associated with adverse mental and physical health (20). Others suggest that the social meaning attached to being black, including factors such as social class differentiation, social stereotypes, or class prejudice, may play a role in health outcomes (18).

The positive association between the percentage of children living in poverty and premature mortality among HIC is consistent with hypotheses surrounding relative status and deprivation. Relative deprivation, which has been associated with negative stress-related behaviors and adverse health outcomes, may differentially affect health across income levels (22) and may actually worsen health in high-income areas (23). Indeed, psychosocial processes related to status, social comparisons, perceptions of inequality (15), or stress resulting from the acceptance of the societal stigma of inferiority (18) may have pathogenic consequences that adversely affect health.

These findings are further supported by the positive association between income inequality and premature mortality in HIC. Greater income inequality may increase status competition and status insecurity and is associated with adverse health outcomes, including mortality (3). Although we did not find an association between income inequality and premature mortality among LIC, research suggests that social capital influences or the overall affluence of an area mediates the effect of income inequality on health among low-income groups (4). Other research suggests that the relationship between income inequality and health is more significant at the national and state level rather than at substate levels (20).

Studies indicate that geography may modify racial/ethnic or social class disparities (22,24). Accordingly, our multivariable analyses found significant regional effects in the low-income and high-income strata. In addition, disparities in quality of health care or government spending on infrastructure resources could also be important. In a county-level examination of US mortality, Singh and Siahpush (25) linked county deprivation with lower local government spending on safety, fire protection, social and welfare services, education, affordable housing, and employment. Evidence also shows that residents in the Northeast are much more likely than those in the Southwest to receive effective health care (24), and, indeed, the protective effects for LIC of being in the Northeast observed here are striking. Further research should explore the possible importance of cultural, political, or religious factors not measured in this study.

The association between higher rates of uninsurance and better mortality outcomes in LIC was surprising. We believe that self-selection processes may be a factor, as suggested in prior research (26). It is possible that in LIC, only those with the greatest health care needs enroll in public health insurance programs such as Medicaid. In turn, this may permit low-income, uninsured people who are healthier than the insured population. Although this may partially account for the association, more research is needed to confirm and explain this finding.

We identified a number of risk factors that were associated with premature mortality rates among US counties at any income level. The significant effects of behavioral factors confirm previous research (27), and the negative association between percentage Hispanic and premature mortality across all income groups supports the "Hispanic paradox," the paradoxical relationship between health and socioeconomic status of Hispanics (28) at the county level. The large effect sizes of college graduation across all income levels underline its importance and should be of interest to policy makers who wish to improve health outcomes.

A limitation of this study was the use of ecologic data to derive associations that might indicate causal relationships. The units of analysis for this study were counties and, thus, interpretations of specific associations between predictor variables and mortality should be made with caution. The "ecologic fallacy" — an assumption that associations among variables assessed from aggregate data apply to analogous individual-level variables — could easily lead to misinterpretation of the model. On the other hand, ecologic analysis is often the method of choice when the unit of analysis is a geographically defined area (eg, a county of a state) where public health action is being considered (29,30). Individual-level analyses often ignore the socioeconomic context within which people experience different levels of health (29). In addition, intervening in one of the modifiable variables may also change the prevalence of other variables, resulting in a lower estimate of effect.

Another limitation was limiting outcomes to premature mortality rates. One would expect different relationships using health-related quality of life indicators or disparity measures; further work should examine these outcomes (31). In addition, even though the dates of the determinant data precede, or in some cases overlap, the period of the mortality measures, many of them have a latency, even long latency, in producing outcomes. Future work should attempt to appropriately lag as many determinant variables over the life course as possible. Further, our choice to examine health on a county level may have concealed important within-county variations, such as neighborhood context. We were also unable to adjust for the effect of migration on the outcome, leaving the impression that the outcomes are associated with local determinants when in fact some of them are the result of exposures in other places.

This study identified large variations in premature mortality rates in both low- and high-income counties of the United States, variables associated with this variation, and differences in the pattern between low- and high-income groups. The magnitude of the associations reinforces a population health perspective that health outcome improvement will require a balanced investment strategy across health care, education, behaviors, and the social environment. Additional research is needed to improve causal understanding of these relationships to guide investment decisions of public and policy makers who wish to reduce these disparities; however, our research suggests that policies aimed at improving population health may be more successful if they reflect variations in social and economic contexts.

## Figures and Tables

**Table 1.** Characteristics of US Counties by Income Group^a^

County Characteristic (Year Assessed)^b^	All Counties (N = 3,139)	County Group^c^		

		Low-Income (n = 785)	Mid-Income (n = 1,570)	High-Income (n = 784)
**Premature mortality rate per 100,000 (2002-2006), mean (SD)**	417 (102)	496 (110)	410 (83)	351 (68)
**Income (2002-2006), mean (SD)**				
Median household income in thousands, $ (1999)	35.4 (8.9)	26.2 (2.9)	34.0 (2.8)	47.8 (8.0)
Income inequality (Gini index^d^) (2005-2007)	43.3 (3.5)	46.1 (3.0)	42.8 (2.9)	41.2 (3.6)
**Region (1999), %^e^**				
Northeast	7	1	7	14
Midwest	34	20	38	38
South	45	70	41	30
West	14	10	14	18
**Population density per square mile (1999), %^e^**				
<17	25	37	27	10
17-42	25	38	27	9
43-103	25	21	27	24
>104	25	4	19	57
**Race/ethnicity (1999), % (95% CI)**				
Non-Hispanic white	87.0 (86.5-87.6)	80.2 (78.7-81.8)	90.0 (89.3-90.6)	88.0 (87.1-88.9)
Non-Hispanic black	9.0 (8.5-9.5)	14.8 (13.3-16.3)	7.2 (6.6-7.7)	6.8 (6.1-7.4)
Nonblack, nonwhite	2.9 (2.6-3.2)	4.4 (3.5-5.3)	2.2 (1.9-2.4)	2.8 (2.3-3.2)
Hispanic	7.0 (6.6-7.5)	8.4 (7.1-9.6)	6.4 (5.9-6.9)	6.9 (6.3-7.5)
**Health care access and quality**				
Uninsured residents (2005), % (95% CI)	18.0 (17.8-18.2)	20.6 (20.2-21.0)	17.8 (17.5-18.1)	15.8 (15.4-16.1)
Preventable hospital stays per 1,000 (2005-2006), mean (SD)	90.6 (36.0)	117.5 (45.6)	87.0 (29.1)	71.6 (18.8)
Primary care providers per 100,000 (2006), mean (SD)	85.3 (59.6)	68.0 (65.1)	85.2 (55.0)	103.0 (57.7)
**Socioenvironmental factors, % (95% CI)**				
High school freshman graduation rate (2005-2006)	78.5 (78.0-78.9)	75.6 (74.6-76.6)	79.0 (78.4-79.6)	80.2 (79.5-80.9)
Adults with a 4-year college degree (2005-2007)	17.8 (17.5-18.1)	13.0 (12.6-13.4)	16.5 (16.2-16.8)	25.1 (24.4-25.8)
Children living below federal poverty guidelines (2007)	21.1 (20.8-21.4)	31.0 (30.5-31.7)	20.2 (20.0-20.5)	12.8 (12.4-13.1)
Single-parent households (2005-2007)	8.8 (8.7-8.9)	9.7 (9.4-10.0)	8.5 (8.4-8.6)	8.5 (8.4-8.7)
**Behavioral factors, % (95% CI)**				
Residents who smoke (2002-2008)	22.4 (22.2-22.7)	24.4 (23.8-25.0)	22.7 (22.4-23.0)	20.5 (20.1-20.9)
Obese residents (2006-2008)	28.3 (28.2-28.4)	30.0 (29.8-30.4)	28.2 (28.1-28.4)	26.6 (26.3-26.8)

Abbreviations: SD, standard deviation; CI, confidence interval.

a Data sources: 2010 County Health Rankings (www.countyhealthrankings.org), Centers for Disease Control and Prevention Compressed Mortality database (http://wonder.cdc.gov/wonder/), and 2010 Decennial Census (www.census.gov/main/www/cen2000.html). Percentages without CIs are reported for region and population density categories. All differences between high-income and low-income characteristics are significant at *P* < .001 except for percentage Hispanic (*P* < .03).

b Defined as all-cause, age-adjusted mortality rate per 100,000 population aged birth to 75 years, averaged from 2002 through 2006.

c Income groups are defined by sample county median household income levels: low-income, <$28,631; mid-income, $29,631-39,401; and high-income, >$39,401. Values may not total 100% because of rounding.

d The Gini index scores income inequality in resources from 0 (complete equality) to 100 (complete inequality).

e Significance determined for overall group difference.

**Table 2.** Multivariable Regression Model of Premature Mortality, 3,139 US Counties, 2002-2006^a^

**County Characteristic**	SD	β (SE)^b^	*P* Value^c^	% Change^d^
**Income **				
Median household income	8.9	−0.02 (0.01)	<.001	−2.7
Income inequality (Gini index^e^)	3.5	0.03 (0.004)	<.001	2.1
**Region**				
Northeast	1	−0.08 (0.01)	<.001	−12.5
Midwest	1	−0.09 (0.01)	<.001	−16.4
South	1	0.04 (0.01)	<.001	−6.1
West	1	1 [Reference]		
**Population density per square mile**				
<17	1	−0.03 (0.02)	.13	−2.3
17-42	1	−0.02 (0.01)	.01	−1.3
43-104	1	−0.01 (0.01)	.06	−0.5
>104	1	1 [Reference]		
**Race/ethnicity**				
% Non-Hispanic white	16.1	1 [Reference]		
% Non-Hispanic black	14.5	0.05 (0.003)	<.001	4.2
% Nonwhite, nonblack	8.0	0.01 (0.004)	<.001	0.8
% Hispanic	12.5	−0.02 (0.004)	<.001	−3.7
**Health care access and quality**				
% Uninsured residents	6.1	0.005 (0.004)	.14	−1.7
Preventable hospital stays per 1,000^f^	36.0	0.04 (0.004)	<.001	3.2
Primary care providers per 100,000	59.6	0.006 (0.005)	.24	2.8
**Socioenvironmental factors**				
High school freshman graduation rate^f^	12.3	−0.02 (0.005)	.001	−2.4
% Adults with a 4-year college degree	8.4	−0.06 (0.004)	<.001	−5.5
% Children living below federal poverty guidelines	9.0	0.02 (0.004)	<.001	1.7
% Single-parent households	2.8	0.01 (0.004)	<.001	1.4
**Behavioral factors**				
% Residents who smoke^f^	5.9	0.07 (0.01)	<.001	6.7
% Obese residents	3.9	0.03 (0.003)	<.001	3.0

Abbreviations: SD, standard deviation; SE, standard error.

a Model adjusted for variables listed in table. The model includes an intercept term. Premature mortality defined as all-cause, age-adjusted mortality rate per 100,000 population aged birth to 75 years, averaged from 2002 through 2006.

b β coefficients for continuous covariates reflect the percentage change in the premature mortality rate associated with each SD change in the predictor.

c Percentage change in premature mortality associated with a 1 SD increase in the covariate (or Northeast, Midwest, South vs West; <17, 17-42, 43-104 vs >104 people per square mile).

d Calculated by using [EXP(Beta)-1]*100.

e The Gini index scores income inequality in resources from 0 (complete equality) to 100 (complete inequality).

f Some counties were missing values for this variable (see Methods for list).

**Table 3.** Multivariable Regression Model of Premature Mortality by Income, 3,130 US Counties, 2002-2006^a^

County Characteristic	SD	County Group^b^					

		Low-Income (n = 785)			High-Income (n = 784)		

		β (SE)	*P* Value^c^	% Change^d^	β (SE)	*P* Value^c^	% Change^d^
**Income**							
Median household income	8.9	0.01 (0.02)	.55	1.2	−0.03 (0.01)	<.001	−2.5
Income inequality (Gini index^e^)	3.5	0.003 (0.01)	.58	0.3	0.03 (0.01)	<.001	2.8
**Region**							
Northeast	1	−0.21 (0.12)	.07	−18.8	−0.10 (0.08)	.19	−9.9
Midwest	1	−0.12 (0.17)	.48	−11.2	−0.19 (0.07)	<.001	−17.3
South	1	0.02 (0.11)	.83	2.3	−0.13 (0.11)	.23	−12.0
West	1	1 [Reference]					
**Population density per square mile**							
<17	1	0.03 (0.02)	.20	2.6	−0.05 (0.04)	.21	−4.5
17-42	1	−0.01 (0.01)	.40	−1.3	−0.02 (0.02)	.49	−1.5
43-103	1	0.01 (0.01)	.46	1.0	0.004 (0.01)	.64	0.05
>103	1	1 [Reference]					
**Race/ethnicity**							
% Non-Hispanic white	16.1	1 [Reference]					
% Non-Hispanic black	14.5	0.03 (0.01)	<.001	2.7	0.06 (0.01)	.001	5.9
% Nonblack, nonwhite	8.0	0.003 (0.005)	.47	0.3	0.03 (0.02)	.10	2.7
% Hispanic	12.5	−0.05 (0.01)	<.001	−4.9	−0.05 (0.01)	<.001	−5.0
**Health care access and quality**							
Uninsured residents, %	6.1	−0.04 (0.01)	<.001	−4.4	−0.0003 (0.01)	.97	0
Preventable hospital stays per 1,000	36.0	0.02 (0.003)	<.001	2.4	0.07 (0.01)	<.001	7.6
Primary care providers per 1,000	59.6	0.01 (0.01)	.03	1.2	0.02 (0.004)	<.001	2.2
**Socioenvironmental factors**							
High school freshman graduation rate	12.3	−0.02 (0.01)	<.001	−2.2	−0.02 (0.01)	.01	−2.1
Adults with a 4-year college degree, %	8.4	−0.04 (0.01)	<.001	−4.3	−0.05 (0.01)	<.001	−5.3
Children living below federal poverty guidelines, %	9.0	0.03 (0.01)	<.001	3.1	0.01 (0.01)	.03	2.4
Single-parent households, %	2.8	0.02 (−.01)	<.001	2.2	0.002 (0.01)	.76	0.2
**Behavioral factors, %**							
Residents who smoke	5.9	0.05 (0.01)	<.001	5.1	0.06 (0.01)	<.001	6.2
Obese residents	3.9	0.004 (0.01)	.56	0.4	0.04 (0.01)	<.001	4.2

Abbreviations: SD, standard deviation; SE, standard error.

a Model adjusted for all categories listed in table and includes an intercept term. Premature mortality defined as all-cause, age-adjusted mortality rate per 100,000 population aged birth to 75 years, averaged from 2002 through 2006.

b β coefficients for continuous covariates reflect the percentage change in the premature mortality rate associated with each SD change in the predictor.

c Calculated by using [EXP(Beta)-1]*100.

d Percentage change in premature mortality associated with a 1 SD increase in the covariate (or Northeast, Midwest, South vs West; <17, 17-42, 43-104 vs >104 people per square mile).

e The Gini index scores income inequality in resources from 0 (complete equality) to 100 (complete inequality).
